# Tick Infestation and Piroplasm Infection in Barbarine and Queue Fine de l’Ouest Autochthonous Sheep Breeds in Tunisia, North Africa

**DOI:** 10.3390/ani11030839

**Published:** 2021-03-16

**Authors:** Médiha Khamassi Khbou, Mariem Rouatbi, Rihab Romdhane, Limam Sassi, Mohamed Jdidi, Aynalem Haile, Mourad Rekik, Mohamed Gharbi

**Affiliations:** 1 Laboratory of Infectious Animal Diseases, Zoonosis and Sanitary Regulation, Institution of Agricultural Research and Higher Education, National School of Veterinary Medicine of Sidi Thabet, Univ. Manouba, Sidi Thabet 2020, Tunisia; 2 Laboratory of Parasitology, Institution of Agricultural Research and Higher Education, National School of Veterinary Medicine of Sidi Thabet, Univ. Manouba, Sidi Thabet 2020, Tunisia; rouatbi.myriam@yahoo.fr (M.R.); rihabromdhaneveto@gmail.com (R.R.); sassilimam@yahoo.fr (L.S.); jdidimohamed79@gmail.com (M.J.); gharbim2000@yahoo.fr (M.G.); 3 International Center for Agricultural Research in the Dry Areas (ICARDA), P.O. Box 5689, Addis Ababa, Ethiopia; a.haile@cgiar.org; 4 International Center for Agricultural Research in the Dry Areas (ICARDA), P.O. Box 950764, Amman 11195, Jordan; m.rekik@cgiar.org

**Keywords:** breed, sheep, resistance, ticks, piroplasms, Tunisia

## Abstract

**Simple Summary:**

Ticks and tick-borne pathogens affect the productivity of sheep in Tunisia. Searching for genetically resistant breeds to infestation by ticks may represent an alternative to the overuse of chemical drugs. The aim of this study was to assess if there is any difference in tick infestation among the main sheep breeds in Tunisia. Four hundred and thirty-nine ear-tagged ewes from Barbarine and Queue Fine de l’Ouest (QFO) breeds were examined and sampled each trimester for two years. Ticks were identified to the species level, and piroplasms were detected using Polymerase Chain Reaction (PCR). Queue Fine de L’Ouest ewes were markedly less infested by ticks, and none was infected by piroplasms compared with Barbarine conterparts. The QFO sheep breed could be considered in concrete control strategies, including a breeding program.

**Abstract:**

As ticks and tick-borne pathogens affect the productivity of livestock, searching for genetically resistant breeds to infestation by ticks may represent an alternative to the overuse of chemical drugs. The aim of this study was to assess if there is a difference in tick infestation among the main sheep breeds in Tunisia. The study was carried out between April 2018 and January 2020 in 17 small to middle-sized sheep flocks from 3 regions across Tunisia. Four hundred and thirty-nine ear-tagged ewes from Barbarine (n = 288, 65.6%) and Queue Fine de l’Ouest (QFO) (n = 151, 34.4%) breeds were examined and sampled each trimester. Ticks were identified to the species level, and piroplasms were detected using PCR that targets a common sequence ARNr18S to both *Babesia* and *Theileria* genera using catch-all primers. Totally, 707 adult ticks were collected from animals; 91.4% (646/707) of them were *Rhipicephalus sanguineus* s.l. Queue Fine de l’Ouest animals were markedly less infested by ticks, and no one of them was infected by piroplasms compared to the Barbarine breed. Indeed, during the first four seasons, 21 animals, all from the Barbarine breed, were detected positive for piroplasms. This is the first study in Tunisia about the low susceptibility of QFO ewes to infestation by ticks and to infection by piroplasms. The QFO sheep breed could be raised preferably at high-risk areas of tick occurrence and could be considered in concrete control strategies, including a breeding program.

## 1. Introduction

Several parasite infections compromise small ruminant health, food security and human health. In Tunisia, sheep face several endoparasites and ectoparasites—when cumulated to bacterial and viral infections and to technically irrational herd management practices—that deeply impede the whole sector development. Ticks and tick-borne pathogens (TBP) (*Theileria* spp., *Babesia* spp., *Anaplasma* spp., *Borrelia* spp.) affect the productivity of sheep and may cause huge economic losses [1,2,3,4]. In addition to the pathogens they transmit, ticks cause skin irritations, dermatitis, bacterial infections and blood spoliation that could lead to chronic anemia [5]. Ticks are conventionally controlled through acaricides application. However, resistance to acaricide is growing and spreading in tick populations, leading to the failure of anti-ticks control programs [6,7]. Moreover, chemical acaricides are toxic to consumers, persons handling animals, treated animals and are persistent in the environment, making such a strategy not complying with the principles of Eco-Health [8]. Searching for genetically resistant breeds of livestock to infestation by ticks may represent an irreversible and sustainable mechanism for the control and an alternative to the overuse of chemical drugs. Although the genetic background is responsible for any inherent resistance mechanism to ticks [9], other factors linked to the animal or its environment such as age, gender, physiological status, behavior, coat characteristics, size, food regimen, as well as climate and environment, could have a role [10]. Resistance to tick infestation was widely investigated in cattle [11,12] and experimental animals such as guinea-pigs [13,14]. In cattle, resistance to ticks was shown to be heritable [15], and in South African cattle, heritability for tick resistance assessed by tick counts was estimated between naught and 0.89 [16]. There are limited studies and reports on sheep. In this important livestock species, the genetic resistance was investigated mainly for flies [17] and gastrointestinal nematodes [18]. The heritability of resistance to ticks was estimated to 0.32 to 0.59 in Norwegian sheep [19], and since heritability for tick resistance is moderate [20], such a trait could be included in breeding programs.

The genetic resistance to tick infestation is also immunologically mediated. Both innate and acquired immunity are involved in tick resistance [21]. Wada et al. [22] reported that resistance to tick appears after repeated tick infestations, the fourth infestation in cattle [23] but after a single tick infestation in sheep [24]. The histological examination of tick site attachment, the quantification of circulating T and B lymphocytes [25], cytokine synthesis [26] and examination of gene expression in the skin [27] were investigated by several authors mainly for tick resistance in cattle, but not in sheep.

Most of the studies on ticks and tick-borne pathogens in Tunisia focused on comparing prevalence according to livestock species, age, geographic areas, but few included variations between breeds. In a study carried out in the district of Siliana (northwest Tunisia), Elati et al. [28] found that 7.3% (103/284) and 16.7% (303/362) of Barbarine and Queue Fine de l’Ouest (QFO) surveyed animals, respectively, were infested by ticks. This study provided preliminary investigations into the presence of a difference in the receptivity of sheep to ticks. However, the survey period was relatively short, and it was geographically very restricted. Our study is based on two of the main sheep types in North Africa, Barbarine and QFO. Depicting forms of resistance among these 2 breeds for ticks and pathogens transmitted by ticks may add valuable information in designing breeding schemes. Extending over a period of 2 years and covering the main areas where these 2 breeds prevail in Tunisia, the current study investigated variability among Barbarine and QFO sheep to infestation by ticks and infection by piroplasms (*Babesia*/*Theileria*).

## 2. Materials and Methods

### 2.1. Study Region and Sampling Design

**Figure 1:**
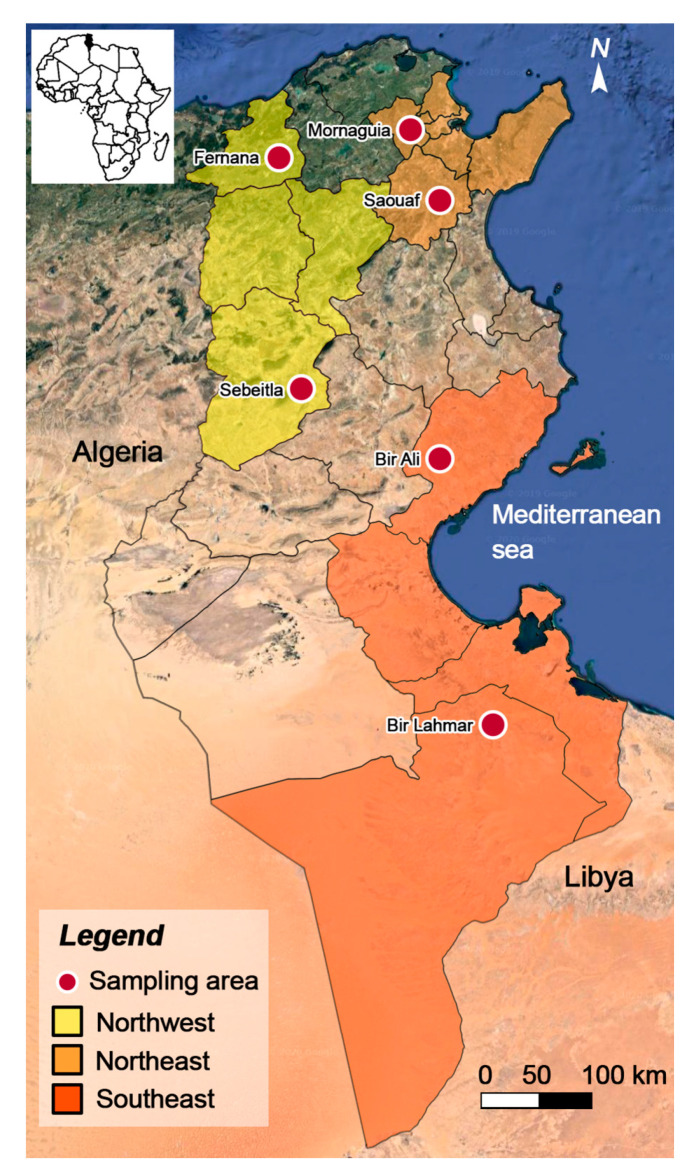
Map of Tunisia showing the sampling areas in the three regions.

This repeated cross-sectional study was carried out from April 2018 to January 2020. A total of 17 small to middle-sized (10 to 50 ewes per flock) and extensively managed sheep flocks were randomly selected from the northeast, the northwest and the southeast regions of Tunisia (Figure 1 and Table 1).

The northeast and the southeast regions are part of the Barbarine sheep homeland even though the breed has a nationwide presence because of its very ancient roots in the country. The northwest region is the main concentration area of the QFO breed [29]. However, last decades have seen an increase in the population of this breed spreading to the central and eastern parts of the country, as a result of the market demand for less fatty meat and also because of the suitability of this breed for more intensive, sedentary type of production [30].

### 2.2. Animals

**Figure 2:**
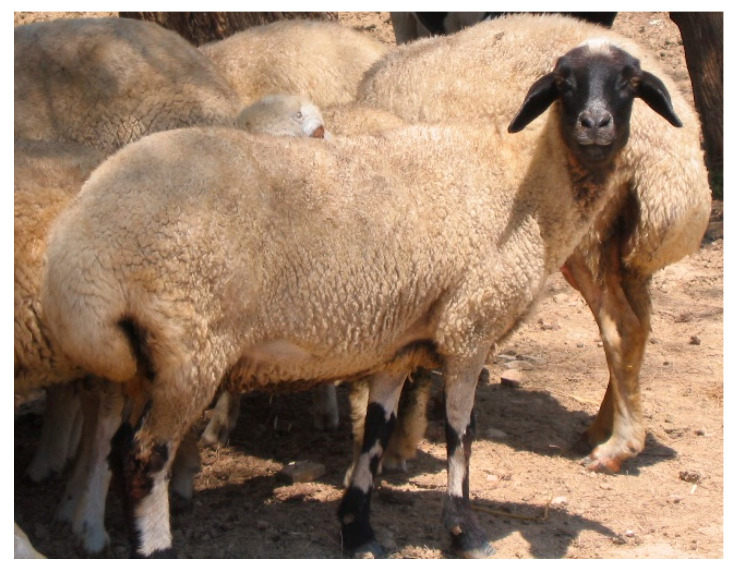
Black-headed Barbarine ewe.

**Figure 3:**
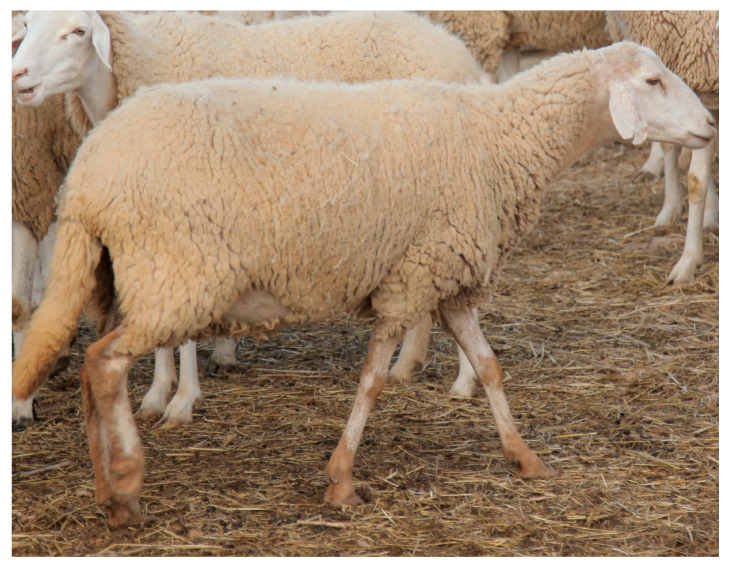
Queue Fine de l’Ouest ewe.

Only yearling and older ewes were included in the present study. Four hundred and thirty-nine ewes were ear-tagged and were monitored at 8 sampling rounds. The breed type was determined based on the description reported by Rekik et al. [29]. The sheep belonged to either the Barbarine (288; 65.6%) or the QFO (151; 34.4%) breeds (Table 1) and were representative of the national distribution of both breeds among the total 4,000,000 female units in Tunisia [31]. Sheep in Tunisia is one of the main sources of red meat in the country (39.3%) (FAO, 2020). The most important sheep breed is the indigenous fat-tailed Barbarine (Figure 2), which represents 64% of the national sheep population. The second most important breed is the Queue Fine de l’Ouest (QFO, Figure 3), known as “Bergui” or “Western Fine Tail”, representing 30% of the existing breeds in the country [32]. The Barbarine breed, locally known as “Nejdi” or “Arab sheep”, was introduced from the steppes of Central Asia by the Phoenicians around 400 Before Christ during the Carthaginian period [33]. It is also the origin of the “Tunis” breed in the USA [34] and the Barbaresca Italian breed [35].

Barbarine sheep are traditionally found in the meridional central and southern steppes of Tunisia; they are also the dominant breed in Libya and expand to eastern Algeria [36]. This sheep breed is generally managed under extensive production systems [37] and is well adapted to the harsh environmental conditions of the country because of the energy reservoir available in the fat tail (1.5 to 7 kg) [38]. This breed is (i) tolerant to both warm and cold climates, (ii) resistant to internal and external parasites, (iii) able to use a wide range of low-quality feed resources (shrubby vegetation, cactus, cereal straw, olive cake, etc.) [36]. Some authors considered that the Barbarine breed is composed of different ecotypes (strains) [39], but genetic studies did not confirm any difference between subpopulations [40].

QFO sheep are genetically very close and often assimilated to the Ouled Jellal (thin-tailed) breed mainly encountered in Algeria, but the breed is also present in the western plateaus of Tunisia and the eastern mixed sheep-crop systems of Morocco [29]. Animals of this breed are reared mainly for meat production [37]. This breed has a relatively good adaptation to harsh and dry environmental conditions, but it remains more sensitive to extreme temperatures than the Barbarine breed [41].

Despite the above-mentioned differences, genetic studies showed a close relationship between Barbarine and QFO breeds, presenting a low genetic diversity or variation (high similarity), thus indicating low heterozygosity levels [32,42]. Authors attributed this observed close relationship to a possible cross migration in the past between these two breeds. Short geographic distances between areas where Barbarine and QFO breeds are distributed may allow this cross migration [43].

In extensive flocks, sheep are reared mixed with goats and/or cows; the presence of horses is limited; however, all sheep flocks own dogs as a guard. Sheep graze year-round on natural rangelands and cereal stubbles in summer. Suckling females are supplemented with concentrate, especially during cold winter. Spring is the main mating season, and most births are distributed between September and February. In this kind of extensive sheep flocks, most farmers do not apply acaricide to prevent ectoparasites, but occasionally they treat against gastrointestinal nematodes. Vaccination campaigns are organized once per year by the National Veterinary Services and consist of preventing Brucellosis, bluetongue disease, foot and mouth disease and sheep-pox disease.

### 2.3. Sampling and Data Collection

During each visit, all the selected ewes were clinically examined for their temperature (fever threshold value: 39.5 °C), the conjunctival mucosa status: anemic (colored in white or light pink), congested (colored in red), normal (colored in pink), the macroscopic aspect of their feces (pasty or solid) and their body score was estimated from poor (score 1) to excellent (score 5) [44]. Five milliliters of blood were collected from each selected animal in sterile vacutainer EDTA tubes via jugular venipuncture.

As in Tunisian sheep, ticks attach mainly in ears [28]; all the present ticks were collected from both ears of each examined animal and counted. In the laboratory, ticks were stored in 70% ethanol until identification under a stereomicroscope according to the key of Walker et al. [45].

### 2.4. Hematology

Hematological analyses were performed with Auto Hematology analyzer BC-2800Vet^®^ (Shenzen Mindray BioMedical Electronics Co., Ltd., Shenzhen, China) for all the blood samples. The hematological study included red blood cell count (RBC) (×10^6^/mL), hemoglobin (Hb) (g/dL) and packed cell volume (PCV) (%). Animals were considered anemic when all the three parameters RBC, Hb and PCV, were below the minimum threshold values 9. 10^12^/L; 9 g/dL and 27%, respectively [46].

### 2.5. Molecular Analysis for Piroplasms Detection

Due to limited resources, we performed molecular analyses for samples collected during the first year: 438, 370, 348, and 321 in April 2018, July 2018, October 2018 and January 2019, respectively. Since animals belong to private farmers, and we did not interfere with the existing management, some animals were not present at subsequent sampling rounds because they were sold, died or transferred to other flocks. Prior to PCR, the DNA was extracted from total blood.

#### 2.5.1. DNA Extraction

The total DNA was extracted from 300 µL anti-coagulated blood using a rapid blood genomic DNA extraction kit (Ref.: BT4782, Bio Basic, Markham, Canada). All the DNA extraction steps were applied as recommended by the manufacturer, except for two steps. Indeed, after adding protein precipitation solution, the samples were placed at −20 °C for 45 min instead of the 20 min recommended by the manufacturer, followed by centrifugation at 16,000× *g* instead of 12,000× *g*. This step was repeated if the obtained pellet still contained protein. Extracted DNA was double-aliquoted and stored at −20 °C until further analyses. The quality of extracted DNA was checked by universal polymerase chain reaction (PCR).

#### 2.5.2. Polymerase Chain Reactions

To verify the presence and the integrity of DNA in each extract, a set of universal primers 1A and 564R (Table 2), targeting simultaneously the 18S and the 16S ribosomal RNAs genes of eukaryotic and prokaryotic organisms, respectively, were used according to the protocol of Wang et al. [47]. This universal PCR was carried out in 25 µL reaction volume consisting of 1× PCR buffer, 2 mM MgCl_2_, 10 µM of each primer, 0.2 mM of each dNTP, 2 U Taq polymerase (Vivantis, Chino, CA, USA), and 1.5 μL of DNA template. The following cycling profile was used: initial denaturation for 5 min at 94 °C, followed by 25 cycles (94 °C, 59 °C and 72 °C for 50 s each) and a final extension at 72 °C for 10 min, and holding temperature of 4 °C at the end of the run.

To detect a common sequence ARNr18S to both *Babesia* and *Theileria* genera, catch-all primers (RLB F and RLB R) (Table 2) were used on samples that were positive in the Universal PCR. Reactions were performed in 25 µL volume containing 19 µL PCR buffer, 1.5 mM MgCl_2_, 200 µM of each deoxyribonucleotide triphosphate, 0.125 µg of Taq hot start Ab, 0.1 U of Uracil DNA glycosylase, 2.5 × 10^−5^ µM of each primer and 1.25 U of Super Taq DNA polymerase (Vivantis, Chino, CA, USA) and 3 µL of sampled DNA [48]. The DNA of pure culture of *Theileria annulata* and sterile water were used as positive and negative controls, respectively. The cycling conditions consisted of an initial denaturation of 5 min at 94 °C, followed by 8 cycles (denaturation at 94 °C for 20 s, followed by annealing at 67 °C for 30 s, and elongation at 72 °C for 30 s). The annealing temperature was decreased by 2 °C every two cycles (from 67 °C to 59 °C). The previous step was followed by 40 cycles (denaturation at 94 °C for 20 s, followed by annealing at 57 °C for 30 s, and elongation at 72 °C for 30 s) and a final extension of 7 min at 72 °C. At the end of the run, the holding temperature was about 4 °C.

The PCR products were examined by electrophoresis on 1.5% agarose gel stained with ethidium bromide and visualized under ultraviolet light. The size of the amplified fragment is about 460–520 pb.

### 2.6. Statistical Analyses

The following epidemiological indicators [49] were estimated:

Tick infestation prevalence (%) = 100 × (Number of infested sheep/Total number of examined sheep)

Mean intensity (m_i_) = number of ticks/total number of infested sheep

Mean abundance (m_a_) = number of ticks/total number of examined animals

The molecular prevalence of *Babesia*/*Theileria* infection (%) was calculated as 100× (number of PCR positive sheep/total number of tested sheep).

For the total period from April 2018 to January 2019, the total number of infected animals is different from the total positive *Babesia*/*Theileria* because each positive animal was counted one time even if found infected more than once.

All data were analyzed using SPSS 21 software (IBM, New York, NY, USA). The 95% confidence intervals for proportions and means were estimated, according to Schwartz [50]. For comparison between proportions in large and small samples, the chi-squared test (χ^2^) and Fisher’s exact test were used, respectively. For the between months comparison of the mean intensity and the mean abundance, one-way ANOVA was performed followed by post hoc Tukey’s test. The correlation between tick counts and hematological parameters was tested by the coefficient of Pearson’s (r). To test the effect of breed on tick infestation prevalence, a binary logistic regression was applied.

As an initial Poisson regression of tick count showed overdispersion, a negative binomial regression model was applied [51]. In this model, the tick count was considered the dependent variable, while both the region and the breed as predictor factors. The interaction between breed and region was also considered in the model. All statistical tests were considered significant at a threshold of 0.05.

### 2.7. Ethics Statement

The animals sampled in this study were owned by private sheep farmers. The sheep owners were aware of the objectives of the study, and the animals were sampled with their permission, in their presence and with the supervision of a qualified veterinarian. The sampling procedures were performed according to the guidelines for the care and use of animals of the National School of Veterinary Medicine, Tunisia. During or after the sampling process, no animal was injured or dead, no female aborted.

## 3. Results

### 3.1. Overall, Collected Tick Population and Overall Parasitological Indicators

During the 8 sampling rounds, all the ticks collected from examined ewes (n = 707) were adults. The dominant tick species was *Rhipicephalus sanguineus* s.l. (91.4%; 646/707) followed by *Hyalomma impeltatum* (4.4%; 31/707), *Hyalomma excavatum* (2.1%; 15/707), *Hyalomma marginatum* (0.7%; 5/707), *Hyalomma dromedarii* (0.6%; 4/707) and *Rhipicephalus annulatus* (0.3%; 2/707) (*p <* 0.001) (Table 3).

During the total period of sampling, almost 45% (317/707) of ticks were collected from the southeast region, whereas 42% (296/707) and 13% (94/707) were collected from the northeast and the northwest, respectively.

The between months distribution of ticks was more important for abundance compared to intensity (Table 4 and Table 5).

The highest and lowest overall tick infestation prevalences occurred in July 2019 (34 ± 2.8) and in January 2019 (1.8 ± 0.7), respectively (*p* < 0.001) (Table 4). The highest mean intensity (92/23; 4) occurred in October 2019, whereas the lowest occurred in October 2018 (20/18; 1.11) (Table 4). The highest (215/288; 0.75 ± 0.09) and lowest (9/341; 0.03 ± 0.01) means’ abundance was estimated in July and January 2019, respectively (Table 4).

**Figure 4:**
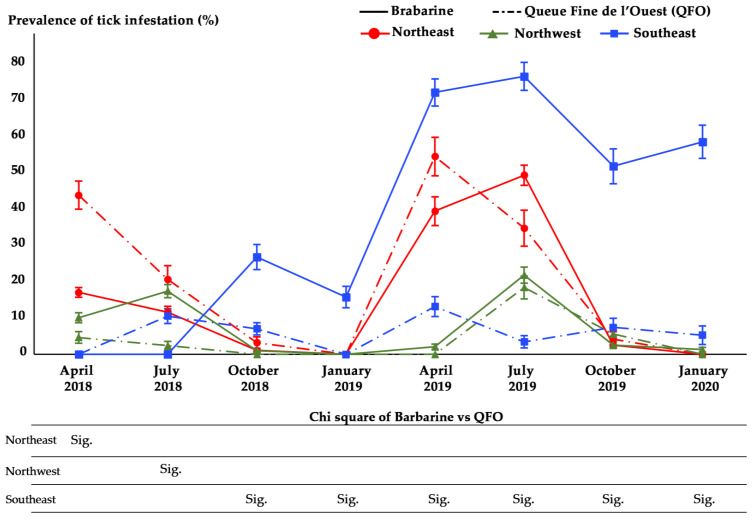
Tick infestation prevalence according to regions and sheep breeds during the eight sampling rounds. Bars: standard errors; Sig.: statistically significant infestation prevalences difference between sheep breeds using the χ^2^ test.

The highest prevalences were recorded in April and July of both 2018 and 2019 in the northeast. During the visits of October and January 2019 and 2020, the highest prevalences were recorded in the southeast (Figure 4, Table S1).

### 3.2. Breed Differences in Tick Infestation

#### 3.2.1. Tick Species Distribution

The majority of ticks were collected from Barbarine when compared to QFO ewes (77.4% and 22.6%, respectively) (*p* < 0.001). More than 70% of the ticks infesting Barbarine sheep were *Rhipicephalus sanguineus* s.l. (Table 3). All the *Hyalomma* spp. tick species (55/707) and one male *Haemaphysalis sulcata* specimen were collected only from Barbarine ewes (Table 3).

#### 3.2.2. Tick’s Infestation Prevalence According to Sheep Breed and Region

**Figure 5:**
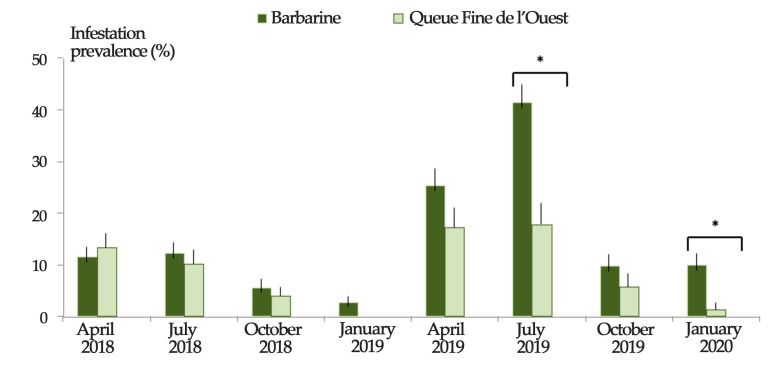
Tick infestation prevalence among two sheep breeds in Tunisia during 8 successive sampling rounds. * Statistically significant infestation prevalences difference between sheep breeds at *p* ≤ 0.05 using χ^2^ test. Bars: standard error.

**Figure 6:**
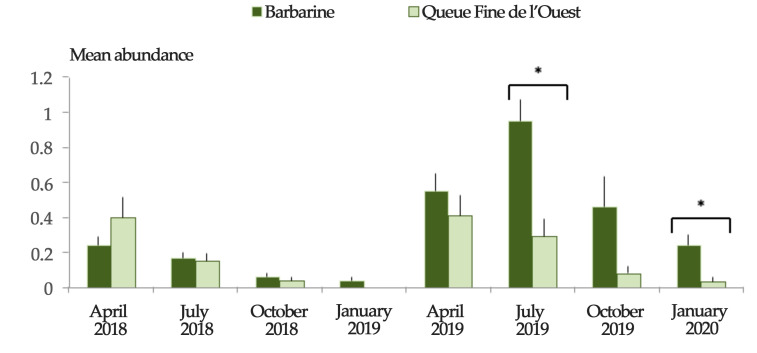
Tick abundance among two sheep breeds in Tunisia during 8 successive sampling rounds. * Statistically significant mean abundance difference between sheep breeds at *p* ≤ 0.05 threshold using χ^2^ test. Bars: standard error.

**Figure 7:**
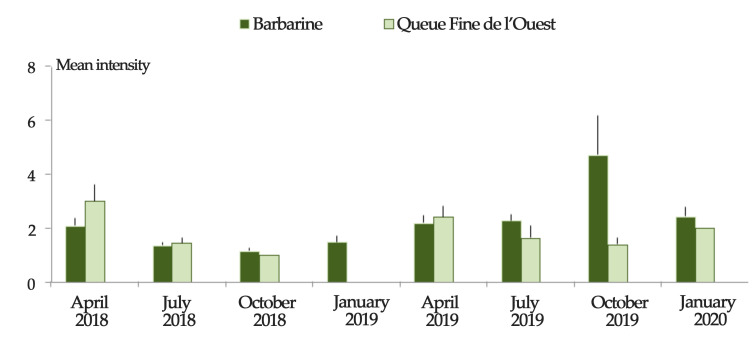
Tick infestation intensity among two sheep breeds in Tunisia during 8 successive sampling rounds. Bars: standard error.

Regardless of the region, Barbarine ewes were more infested than QFO ewes (*p* ≤ 0.05) in July 2019 and January 2020 (Figure 4 and Figure 5), with the same trend for mean abundance (Figure 6). The mean intensity was higher for Barbarine sheep during all the sampling months of 2019 and 2020, except for April (Figure 7).

Taking the regions into account, Barbarine ewes from the southeast showed significantly higher infestation prevalence than QFO breed starting from October 2018 and the subsequent sampling rounds (Figure 4, Table S1). In the northwest region, the same trend was only noted in July 2018 when prevalences of 17.4% (19/109) and 2.4% (1/42) were recorded for Barbarine and QFO, respectively. In the northeast, the estimated prevalence of infestation was significantly higher in QFO than Barbarine ewes only during April 2018, with 44% (18/41) and 17% (21/123), respectively (Figure 4, Table S1). When using a binary logistic regression, the QFO sheep had less risk (odds ratio = 0.58; 95% CI: (0.44–0.78)) to be infested by ticks than the Barbarine sheep breed.

As demonstrated by the negative binomial regression model, “breed”, “region,” and the interaction “region × breed” factors explained the tick count in July 2018, April and July 2019 (Table 6).

### 3.3. Molecular Prevalence of Piroplasms

The overall molecular prevalence in tested ewes was estimated at 2.37 ± 0.4% (35/1477); 21 ewes were infected at least once during the whole period. Eleven sheep out of the 21 were infected during one season, while 6, 3 and 1, were infected in two, three and four successive seasons, respectively. All the infected sheep (n = 21) were from the Barbarine breed; no piroplasms were detected in QFO ewes during the whole sampling period (Table 7). The highest molecular prevalence occurred during October 2018 (13/2019), while in April 2018 and January 2019, only 2 and 8 ewes were infected, respectively. The infection prevalence with piroplasms was different between the three regions, and when taking the sampling rounds altogether, 14.6% (24/164), 5% (8/161) and 2.7% (3/113) were recorded from the northeast, northwest and southeast, respectively (*p* ≤ 0.05).

The percentage of ewes with anemia ranged between 0.44% (2/453) in April 2018 to 3.04% (11/362) in July 2018 (*p* = 0.01). There was no significant correlation between anemia and tick count, positivity to piroplasms, age, and breeds for all the sampling rounds.

## 4. Discussion

The current study highlights that the Queue Fine de l’Ouest breed showed a marked lower tick infestation and no piroplasms infection, compared to Barbarine ewes. This may be an indicator of the existence of a form of genetic variability among sheep breeds in Tunisia with regards to infestation by ticks and TBP. The very low infestation of QFO sheep by ticks between July 2018 and January 2020 and the absence of any animal of this breed infected with piroplasms should be confirmed using experimental tick infestation protocol on larger animals’ samples.

Sheep in Tunisia are facing several pathogens that hamper the development of the whole small ruminant sector and impede the small farmers to access the market [52]. Several parasites affect sheep health and induce high economic losses either through abortion such as *Toxoplasma gondii* [53] and *Neospora caninum* [54] or through general health deterioration caused by gastrointestinal nematodes [55], lungworms [56] and tick-borne pathogens.

### 4.1. Breed Differences

Available reports on sheep tick infestation in Tunisia are very scanty for any consistent comparisons to be made between breeds. A study by Elati et al. [28] indicated that Barbarine sheep were more resistant to tick infestation than QFO animals, which is contradictory to our findings. Indeed, the results of Elati et al. [28] were not representative of the two breeds in the country and their dominant management systems because the sampling was limited to large flocks under intensive management in one single location in northwest Tunisia.

We, therefore, anticipate that the results obtained in our study reflect much better differences between the two breeds because (i) both breeds were sampled in their actual areas of expansion in the country, (ii) targeted flocks had a small to medium size under an extensive management system which is typical in the country [29], and (iii) the repeated frequency of sampling over a period of almost 2 years pleads towards better representativeness and quality of the obtained data.

Moreover, in the southeast region, where almost 45% of total ticks were collected, and during six sampling rounds (from October 2018 to January 2020), Barbarine ewes were most infested than QFO ewes. Those animals of both Barbarine and QFO breeds were also kept under the same husbandry conditions, and the difference of infestation between breeds is less likely to be due to confounding factors.

Tick resistance was intensively investigated in cattle, and it was shown that zebu cattle (*Bos indicus*) were more resistant than taurine cattle (*Bos taurus*) [57,58,59,60]. Both cellular and humoral reactions of zebu infested by ticks confirmed these observations [26,61,62]. Such evidence led to the successful selection of tick resistant cattle as part of cattle-tick control schemes in Australia, where the *Rhipicephalus australis* is economically the most damaging bovine ectoparasite [63]. In sheep, few studies were carried out to investigate breed differences to tick infestation. Mirkena et al. [64] reported that local sheep breeds were more resistant in general to stressors such as ticks. Cloete et al. [65,66] argued that the local South African Namaqua Afrikaner fat-tailed breed outperformed the commercial Dorper and South African Mutton Merinos breeds in terms of low tick counts. The heritability of “total tick count” was estimated to be 0.39–0.54 [19] and 0.43–0.44 [66] among Norwegian lambs and South African sheep breeds, respectively. Resistance to ticks is dependent on the tick species, the host and the environment. It could be measured by estimating the reduction in the numbers of ticks, particularly the engorged specimens decrease, the weight of engorged ticks and the duration of blood-feeding besides interruption of tick development [57]. These indicators are suitable to be measured when animals are kept under experimental conditions.

Queue Fine de l’Ouest ewes were also less susceptible to the infection by piroplasms, but further studies are needed to confirm if this low susceptibility is not due to low tick infestation occurrence or to other factors. Previous descriptive studies carried out in Tunisia support our findings and showed that QFO breed was always less infected than Barbarine breed by several tick-borne pathogens: *Babesia ovis*, *Theileria ovis*, *Mycoplasma ovis*, *Anaplasma ovis* and *Borrelia burgdorferi* s.l. [2,4,67,68].

### 4.2. Sheep Ticks and Agroecological Variability

The majority (646/707, 91.4%) of collected ticks during the 8 successive seasons were *Rhipicephalus sanguineus* s.l., the most widespread tick in the world that colonizes both temperate and tropical regions [69]. Almost two-thirds (75.4%) of the *Rhipicephalus sanguineus* ticks were collected from Barbarine ewes, while 24.6% were collected from QFO ewes. Further studies are needed to confirm resistance to *Rhipicephalus sanguineus* among QFO sheep in Tunisia. *Hyalomma impeltatum* and *Hyalomma dromedarii* were found exclusively in the southeast, they are adapted to the Saharan climate, and they also infest dromedaries (data not shown). *Hyalomma* spp. ticks are very important vectors since they transmit the Crimean Congo hemorrhagic fever virus (CCHFv) [70]. As sheep play an important role as reservoir of CCHFv and several other tick-borne viruses, it could be of paramount interest to select tick-resistant sheep breeds to reduce the infection of these animals.

*Hyalomma excavatum* was mostly found in the southeast, and this is concordant with the findings of Rjeibi et al. [71], who reported that 84.3% (118/140) of this tick species were collected from sheep in the southwest of Tunisia (Kébili district). Since Rjeibi et al. carried out the survey only in QFO sheep breed, no comparison was made with other sheep breeds regarding tick infestation. *Hyalomma excavatum* was also collected from Barbarine sheep in Sened locality (Gafsa district, Center-west Tunisia), an arid region with the maximum temperature reaching 49 °C in summer [72].

### 4.3. Dynamic of Sheep Ticks under Climate Change Context

The dynamic activity of *Rhipicephalus sanguineus* along the 8 successive trimesters was marked by an important peak in summer (July 2019) and the smallest one in spring (April 2018 and 2019). Our results are consistent with the basic knowledge on *Rhipicephalus* spp. ticks activity, which is found along the year with a peak of activity in spring and summer [69,73]. This is also concordant with the findings of Elati [28] and Rjeibi [67], in which the highest values of tick infestation prevalence were observed in August and July, respectively. According to Köppen–Geiger classification, the climate in Tunisia is classified as the Mediterranean with hot summer in the northern part, whereas it is classified semi-arid in Central Tunisia, hot in center-east and cold in center-west and desert in south [74]. These variabilities in climate patterns from north to south of Tunisia explain the polymorphism of tick parasitism on sheep in different Tunisian regions. Indeed, the average minimum temperatures in January ranged between 2.6 to 6.8 °C [75] in north Tunisia, and ticks are not found on animals, contrary to south Tunisia, where ticks were present during the same period. All the tick infestation parameters increased during the visits of 2019 and 2020 in comparison to 2018. The observed difference could be explained by the warmest temperatures recorded during 2019. Indeed, the World Meteorological Organization [76] announced that 2019 was the 2nd warmest year in the last decade, with a 1.5 °C increase in mean temperature. Based on the calculation of the regional climate change index (RCCI), the Mediterranean region appeared as the primary hotspot, making the Mediterranean countries, including Tunisia, vulnerable and the most prone to climate change [77]. As climate change is incriminated in the increase of several ticks and tick-borne pathogens burdens [78], it is expected that tick distribution will change in Tunisia in the near future.

## 5. Conclusions

The Tunisian Queue Fine de l’Ouest sheep breed appears to present an interesting phenotypic resistance to ticks and piroplasms when compared to the Barbarine breed, even when kept under the same herd’s management and environmental conditions. This finding needs more investigations from genetic/genomic, immunologic and behavioral perspectives to fully understand mechanisms of resistance and make concrete control strategies.

## Figures and Tables

**Table 1:** Characteristics of the surveyed sheep flocks.

Region	Locality (District)	Number of Surveyed Farms (Total Number of Animals)	Number of Barbarine Sheep in the Region/Total Number of Barbarine Sheep (%)	Number of QFO Sheep in the Region/Total Number of QFO Sheep (%)
Northeast	Mornaguia (Manouba) and Saouef (Zaghouan)	6 (164)	123/288 (42.7)	41/151 (27.1)
Northwest	Fernana (Jendouba) and Sebeitla (Kasserine)	6 (162)	119/288 (41.3)	43/151 (28.5)
Southeast	Bir Ali (Sfax) and Bir Lahmar (Tataouine)	5 (113)	46/288 (16)	67/151 (44.4)
Overall		17 (439)	288 (100)	151 (100)

QFO: Queue Fine de l’Ouest breed.

**Table 2:** Primers used for universal and piroplasms detection PCRs.

Primers	Sequences (5′------3′)	Author
1A	AACCTGGTTGATCCTGCCAGT	[47]
564R	GGCACCAGACTTGCCCTC	
RLB-F	GAGGTAGTGACAAGAAATAACAATA	[48]
RLB-R	TCTTCGATCCCCTAACTTTC	

**Table 3:** Distribution according to sheep breed of tick species collected during 8 sampling rounds.

Tick Species	Number of Ticks in the Breed (%) ^a^		
	Barbarine	Queue Fine de l’Ouest	Overall (%)
*Rhipicephalus sanguineus* s.l.	487 (75.4)	159 (24.6)	646 (91.4) ^b^
*Hyalomma impeltatum*	31	0	31 (4.4)
*Hyalomma excavatum*	15	0	15 (2.1)
*Hyalomma marginatum*	5	0	5 (0.7)
*Hyalomma dromedarii*	4	0	4 (0.6)
*Rhipicephalus bursa*	2	1	3 (0.4)
*Rhipicephalus annulatus*	2	0	2 (0.3)
*Haemaphysalis sulcata*	1	0	1 (0.1)
Overall (%)	547 (77.4) ^c^	160 (22.6)	707 (100)

^a^ % not calculated for denominator <35; ^b^ Overall, percentages between the different tick species are significantly different at *p* ≤ 0.001 using χ^2^ test; ^c^ Overall, percentages between sheep breeds are significantly different at *p ≤* 0.001 using χ^2^ test.

**Table 4:** Mean temperature and cumulative precipitation of the 15 days before tick collections in the three regions and corresponding overall tick infestation prevalence, intensity and abundance during the 8 sampling rounds.

Variables	April 2018	July 2018	October 2018	January 2019	April 2019	July 2019	October 2019	January 2020	*p*
**Northeast**									
Mean temperature (°C) (Min–Max) ^a^	15.05 (9.7–207)	27.34 (21.09–34.25)	21.48 (17.2–26.73)	10.11 (6.44–14.04)	13.58 (8.93–18.86)	27.96 (21.65–34.32)	21.61 (16.68–27.03)	9.87(6.12–13.99)	N.A.
Cumulative precipitation (mm) ^b^	16.44	0.5	8.02	17.12	44.32	1.58	19.39	14.43	
**Northwest**									
Mean temperature (°C) (Min–Max)	12.48 (7.31–18.23)	22.66 (17.43–30.53)	20.21 (15.93–25–45)	8.02 (3.91–11.27)	11.17 (6.81–15.99)	27.48 (20.72–33.99)	20.80 (15.04–27.07)	9.71 (6.36–13.16)	N.A.
Cumulative precipitation (mm)	40.44	4.22	43.99	33.06	56.06	1.73	15.37	17.5	
**Southeast**									
Mean temperature (°C) (Min–Max)	17.62 (12.12–23.35)	28.7 (22.69–35.26)	23.55 (19.11–28.71)	10.97 (13.07–15.52)	15.05 (11.06–19.57)	30.9 (24.88–37.80)	24.4 (19.9–29.63)	10 (6.83–13.52)	N.A.
Cumulative precipitation (mm)	6	1.6	19.8	5	27.17	0	14.08	16.93	
Infestation prevalence ^c^	53/439	44/382	18/362	6/341	60/269	98/288	23/272	19/258	<0.001 ^f^
(% ± SE)	(12.1 ± 1.6)	(11.5 ± 1.6)	(5 ± 1.1)	(1.8 ± 0.7)	(22.3 ± 2.5)	(34 ± 2.8)	(8.5 ± 1.7)	(7.4 ± 1.6)	
Mean intensity ^d^	129/53	61/44	20/18	9/6	135/60	215/98	92/23	46/19	<0.001 ^g^
(m_i_ ± SE)	(2.43 ± 0.2)	(1.39 ± 0.09)	(1.11 ± 0.07)	(1.5 ± 0.22)	(2.25 ± 0.22)	(2.19 ± 0.19)	(4 ± 1.16)	(2.42 ± 0.3)	
Mean abundance ^e^	129/439	61/382	20/362	9/341	135/269	215/288	92/272	46/258	<0.001 ^h^
(m_a_ ± SE)	(0.29 ± 0.05)	(0.16 ± 0.02)	(0.06 ± 0.01)	(0.03 ± 0.01)	(0.5 ± 0.07)	(0.75 ± 0.09)	(0.34 ± 0.11)	(0.18 ± 0.04)	

SE: standard error; ^a,b^ The mean temperature and the cumulative precipitation were estimated for the 15 days before the tick collection date in each region (crude data were extracted from https://developers.google.com/earth-engine/datasets); N.A.: not applicable; ^c^ number of infested sheep/total number of examined sheep; ^d^ number of collected ticks/Total number of infested sheep; ^e^ number of collected ticks/total number of examined sheep; ^f^ the *p* value was estimated using χ^2^ test to compare the infestation prevalences between the 8 sampling rounds; ^g,h^ the *p* values were estimated by using ANOVA test and Tukey’s test to compare the means between the 8 sampling rounds.

**Table 5:** Multiple comparisons of the mean tick abundance and mean tick intensity according to the eight sampling rounds using the Tukey’s test.

	April 2018	July 2018	October 2018	January 2019	April 2019	July 2019	October 2019	January 2020
**Mean Tick Abundance**								
April 2018	1	0.35	0.009	0.003	0.08	0.001	1	0.61
July 2018	-	1	0.87	0.67	0.001	0.001	0.47	1
October 2018	-	-	1	1	0.001	0.001	0.028	0.87
January 2019	-	-	-	1	0.001	0.001	0.011	0.7
April 2019	-	-	-	-	1	0.445	0.196	0.001
July 2019	-	-	-	-	-	1	0.001	0.001
October 2019	-	-	-	-	-	-	1	0.69
January 2020	-	-	-	-	-	-	-	1
**Mean Tick Intensity**								
April 2018	1	0.15	0.27	0.96	1	0.96	0.15	1
July 2018	-	1	1	1	0.24	0.54	0.001	0.68
October 2018	-	-	1	1	0.37	0.64	0.002	0.65
January 2019	-	-	-	1	0.98	0.99	0.24	0.98
April 2019	-	-	-	-	1	0.99	0.08	1
July 2019	-	-	-	-	-	1	0.009	1
October 2019	-	-	-	-	-	-	1	0.32
January 2020	-	-	-	-	-	-	-	1

Significant values using one-way ANOVA followed by Tukey’s test are *p* ≤ 0.05.

**Table 6:** P values from the negative binomial regression model to test the tick count as a dependent variable with the region, breeds, and their interaction as predictors.

	April 2018	July 2018	October 2018	January 2019	April 2019	July 2019	October 2019	January 2020
Region								
NE	N.S.	N.S.	N.S.	N.S.	0.001	0.003	N.S.	N.S.
NW	N.S.	N.S.	<0.001	N.S.	<0.001	N.S.	N.S.	<0.001
SE ^R^								
Breed								
Barbarine	N.S.	<0.001	0.02	N.S.	<0.001	<0.001	<0.001	0.001
QFO ^R^								
Region × Breed								
NE × Barbarine	N.S.	<0.001	N.S.	N.S.	<0.001	0.001	0.01	N.S.
NE × QFO ^R^								
NW × Barbarine							<0.001	
NW × QFO ^R^								
SE × Barbarine								
SE × QFO ^R^								

^R^: Reference modality; QFO: Queue Fine de l’Ouest sheep breed; NE: northeast; NW: northwest; SE: southeast; N.S.: not significant; empty cells: not applicable for the reference modality and when the value of B = 0).

**Table 7:** Breed differences for molecular prevalence to *Theileria*/*Babesia* during four consecutive sampling rounds.

Sampling Round	Number of *Theileria*-*Babesia* Positive Sheep/Number of Examined Sheep (% ± SE)	
	Barbarine	Queue Fine de L’Ouest
April 2018	2/286 (0.7 ± 0.5) ^a^	0/152 (0) ^b^
July 2018	12/242 (4.96 ± 1.4)	0/128 (0)
October 2018	13/219 (5.94 ± 1.6)	0/129 (0)
January 2019	8/208 (3.85 ± 1.3)	0/113 (0)
Total	35/955 (3.66 ± 0.6)	0/522 (0)

SE: standard error; ^a^ prevalence of tick infestation between seasons within the same column are statistically different at *p* ≤ 0.01 using χ^2^ test; ^b^ Statistics not applicable.

## Supplementary Materials

The following are available online at https://www.mdpi.com/2076-2615/11/3/839/s1, Table S1: Prevalence of tick infestation according to studied regions and sheep breeds during 8 sampling rounds.
