# Supplementary material for: Tick Infestation and Piroplasm Infection in Barbarine and Queue Fine de l’Ouest Autochthonous Sheep Breeds in Tunisia, North Africa
Source: Animals (Basel). 2021 Mar 16;11(3):839. doi: 10.3390/ani11030839 (PMC8001609; doi:10.3390/ani11030839)
Supplement: Supplementary file 1 [file animals-11-00839-s001.pdf]

Table S1. Prevalence of tick infestation according to studied regions and sheep breeds during 8 sampling rounds.

		Number of infested sheep/Total examined (% ± SE)							
**Location**	**Breeds**	**April 2018**	**July 2018**	**October 2018**	**January 2019**	**April 2019**	**July 2019**	**October 2019**	**January 2020**
Northeast	Barbarine	21/123 (17 ± 3.4) [b]	12/103 (11.7 ± 3.2)	1/92 (1.1 ± 1.1)	0/86 (0)	15/38 (39.5 ± 7.9)	40/81 (49.4 ± 5.6)	2/78 (2.6 ± 1.8)	0/76 (0)
	QFO	18/41 (44 ± 7.8)	6/29 (20.7 ± 7.5)	1/31 (3.2 ± 3.2)	0/27 (0)	12/22 (54.5 ± 10.6)	8/23 (34.8 ± 9.9)	1/24 (4.2 ± 4.1)	0/20 (0)
	Total Northeast	39/164 (23.8 ± 3.3) [a]	18/132 (13.6 ± 3)	2/123 (1.6 ± 1.1) [a]	0/113 (0) [a]	27/60 (45 ± 6.4) [a]	48/104 (46.2 ± 4.9) [a]	3/102 (2.9 ± 1.7) [a]	0/96 (0) [a]
Northwest	Barbarine	12/119 (10.1 ± 2.8)	19/109 (17.4 ± 3.6) [b]	1/100 (1 ± 0.9)	0/97 (0)	2/96 (2.1 ± 1.5)	19/87 (21.8 ± 4.4)	2/80 (2.5 ± 1.7)	1/78 (1.3 ± 1.3)
	QFO	2/43 (4.7 ± 3.2)	1/42 (2.4 ± 2.3)	0/41 (0)	0/41 (0)	0/39 (0)	7/38 (18.4 ± 6.3)	2/36 (5.6 ± 3.8)	0/36 (0)
	Total Northwest	14/162 (8.6 ± 2.2)	20/151 (13.2 ± 2.8)	1/141 (0.7 ± 0.7)	0/138 (0)	2/135 (1.5 ± 1)	26/125 (20.8 ± 3.6)	4/116 (3.4 ± 1.7)	1/114 (0.9 ± 0.9)
Southeast	Barbarine	0/116 (0)	0/42 (0) [b]	11/41 (26.8 ± 6.9) [b]	6/38 (15.8 ± 5.9) [b]	26/36 (72.2 ± 7.5) [b]	23/30 (76.7 ± 7.7) [b]	14/27 (52 ± 9.6) [b]	17/29 (58.6 ± 9.1) [b]
	QFO	0/67 (0)	6/57 (1.05 ± 4.1)	4/57 (7 ± 3.4)	0/52 (0)	5/38 (13.2 ± 5.5)	1/29 (3.4 ± 3.4)	2/27 (7.4 ± 5)	1/19 (5.3 ± 5.1)
	Total Southeast	0/113 (0)	6/99 (6.1 ± 2.4)	15/98 (15.3 ± 3.6)	6/90 (6.7 ± 2.6)	31/74 (41.9 ± 5.7)	24/59 (40.7 ± 6.4)	16/54 (29.6 ± 6.2)	18/48 (37.5 ± 7)
Total breeds	Barbarine	33/288 (11.5 ± 1.9)	31/254 (12.2 ± 2.1)	13/233 (5.6 ± 1.5)	6/221 (2.7 ± 1.1)	43/170 (25.3 ± 3.3)	82/198 (41.4 ± 3.5) [b]	18/185 (9.7 ± 2.2)	18/183 (9.8 ± 2.2) [b]
	QFO	20/151 (13.2 ± 2.8)	13/128 (10.2 ± 2.7)	5/129 (3.9 ± 1.7)	0/120 (0)	17/99 (17.2 ± 3.8)	16/90 (17.8 ± 4)	5/87 (5.7 ± 2.5)	1/75 (1.3 ± 1.3)
Total		53/439 (12.1 ± 1.6)	44/382 (11.5 ± 1.6)	18/362 (5 ± 1.1)	6/341 (1.8 ± 0.7)	60/269 (22.3 ± 2.5)	98/288 (34 ± 2.8)	23/272 (8.5 ± 1.7)	19/258 (7.4 ± 1.6)

SE: standard error; QFO: Queue Fine de l'Ouest breed; [a] Percentages between regions within the same column are statistically significant at $p \leq 0.001$ using χ^2 test; [b] Percentages between breeds within the same column are statistically significant at $p \leq 0.05$ using χ^2 test.
